# Prospective Evaluation of Cardiopulmonary Resuscitation Performed in Dogs and Cats According to the RECOVER Guidelines. Part 2: Patient Outcomes and CPR Practice Since Guideline Implementation

**DOI:** 10.3389/fvets.2019.00439

**Published:** 2019-12-10

**Authors:** Sabrina N. Hoehne, Kate Hopper, Steven E. Epstein

**Affiliations:** ^1^William R. Pritchard Veterinary Medical Teaching Hospital, School of Veterinary Medicine, University of California, Davis, Davis, CA, United States; ^2^Department of Veterinary Surgical and Radiological Sciences, School of Veterinary Medicine, University of California, Davis, Davis, CA, United States

**Keywords:** cardiopulmonary resuscitation, guidelines, cardiac arrest, patient outcomes, dog, cat

## Abstract

Cardiopulmonary resuscitation (CPR) outcomes have not been prospectively described since implementation of the Reassessment Campaign on Veterinary Resuscitation (RECOVER) guidelines. This study aimed to prospectively describe CPR outcomes and document arrest variables in dogs and cats at a U.S. veterinary teaching hospital since implementation of the RECOVER guidelines using the 2016 veterinary Utstein-style CPR reporting guidelines. One-hundred and seventy-two dogs and 47 cats that experienced cardiopulmonary arrest (CPA) underwent CPR following implementation of the RECOVER guidelines and were prospectively included. Supervising clinicians completed a data form for CPR events immediately following completion of CPR from December 2013 to June 2018. Seventy-five (44%) dogs and 26 (55%) cats attained return of spontaneous circulation (ROSC), 45 dogs (26%) and 16 cats (34%) had ROSC ≥ 20 min, 13 dogs (8%) and 10 cats (21%) were alive 24 h after CPR, and 12 dogs (7%) and 9 cats (19%) survived to hospital discharge. The most common cause of death in animals with ROSC ≥ 20 min was euthanasia. Patient outcomes were not significantly different since publication of the RECOVER guidelines except for a higher feline survival to hospital discharge rate. Dogs (*p* = 0.02) but not cats with initial shockable rhythms had increased rates of ROSC while the development of a shockable rhythm during CPR efforts was not associated with ROSC (*p* = 0.30). In closed chest CPR an end-tidal carbon dioxide (EtCO_2_) value of >16.5 mmHg was associated with a 75% sensitivity and 64% specificity for achieving ROSC. Since publication of the RECOVER guidelines, CPR practice did not clinically significantly change at our institution and no improvement of already high ROSC rates was noted. The percentage of cats surviving to hospital discharge was higher than previously reported and the reason for this improvement is not evident with these results. Euthanasia remains a major confounding factor in assessing intermediate and long-term CPR outcomes in dogs and cats.

## Introduction

In 2012, the Reassessment Campaign on Veterinary Resuscitation (RECOVER) initiative responded to the growing recognition of the importance of veterinary cardiopulmonary resuscitation (CPR) and published evidence-based consensus guidelines for the performance of CPR in dogs and cats (1). These guidelines not only paved the way for a more uniform approach to veterinary CPR training and performance, but also identified many remaining knowledge gaps surrounding patient population, CPR outcomes, hospital, and arrest variables.

The two largest prospective U.S. studies describing the outcome of dogs and cats suffering from cardiopulmonary arrest (CPA) to date were published in 2009 and 2014 and data for both studies was collected prior to the publication of the RECOVER guidelines (2, 3). A recent Japanese study was able to demonstrate a significant increase in return of spontaneous circulation (ROSC) rates in dogs from 17 to 43% when CPR was performed according to the RECOVER guidelines (4). ROSC rates at U.S. veterinary centers have reportedly ranged from 28 to 60% for dogs and 42 to 57% for cats prior to publication of the RECOVER guidelines (2, 3, 5–7). It is unknown whether the implementation of the RECOVER guidelines also led to a further increase in ROSC rates and survival to hospital discharge in the United States.

Utstein-style guidelines on uniform reporting of in-hospital cardiopulmonary arrest (IHCA) and CPR in dogs and cats were released by the RECOVER initiative in 2016 (8). These veterinary guidelines are part of an effort to standardize terminology when reporting CPA and CPR related events and to make patient population characteristics and patient outcomes comparable across different centers. In human medicine, reporting guidelines for CPR event data were first published in 1991 for the characterization of patients suffering from out-of-hospital cardiopulmonary arrest (OHCA) and since then have been expanded for IHCA, CPA after major trauma, pediatric CPR, CPR research, and post-cardiac arrest care (9–15). The release of veterinary Utstein-style CPR reporting guidelines has only recently made uniform reporting of veterinary CPR possible and to date, only one prospective veterinary study employed their terminology (16).

The main purpose of this study was to prospectively describe CPR outcomes and document arrest variables in dogs and cats at a U.S. veterinary medical teaching hospital since implementation of the RECOVER guidelines using the 2016 veterinary Utstein-style CPR reporting guidelines (8). The secondary aim of this study was to compare these parameters to a cohort from the same facility that received CPR prior to publication of the RECOVER guidelines.

## Materials and Methods

All dogs and cats that suffered from CPA and underwent CPR during the time period from December 2013 through June 2018 were prospectively enrolled in this study. During the study period, CPR was conducted by rescuers trained in CPR according to the RECOVER clinical guidelines for veterinary CPR (1) with the exception of timing of vasopressor and atropine administration as outlined in our accompanying manuscript. Our institutional protocol is to administer vasopressors and atropine as soon as vascular access is obtained without waiting for electrocardiogram (ECG) interpretation. All CPR was carried out at a U.S. university veterinary medical teaching hospital. In our facility, CPR is most commonly conducted in the emergency room, the intensive care unit (ICU), or in operating rooms, all of which have the infrastructure allowing performance of CPR according to the RECOVER guidelines. House officers, faculty clinicians and students undergo didactic and simulation-based CPR training at the start of employment as well as regular refresher sessions. Veterinary nursing staff receive CPR training at the beginning of employment and refresher sessions are provided every 6 months, although attendance is not mandatory.

Immediately upon completion of CPR efforts, during the study period, the supervising clinician filled out a purpose made data form for each CPR event. The same data recording form was used for the entire time of the study. It was created in 2013 based on Utstein-style definitions and reporting templates for human CPR (17). Data recorded on the form included three sets of information surrounding the CPR event; animal, arrest, and outcome variables.

All *animal* core variables, except for chest conformation, were recorded as required by the veterinary Utstein-style CPR reporting guidelines (8). Information regarding all supplemental animal variables (such as disease category at hospital admission or comorbidities at the time of CPA) were also collected on the data form (8). All core and supplemental *arrest* variables were recorded on the data sheet as required by the veterinary Utstein-style CPR reporting guidelines (8). The times from CPA to CPR in animals that arrived at the hospital dead on arrival (DOA) were estimated based on information the owner provided and for patients with an unwitnessed IHCA arrest were estimated based on the last time patient observations were recorded.

All core *outcome* variables were recorded according to the veterinary Utstein-style CPR reporting guidelines. “Any ROSC” was defined as clinical signs of reinstituted effective circulation such as a palpable pulse, systolic blood pressure measurement >60 mmHg in the presence of a direct arterial blood pressure measurement waveform, or a marked increase in end-tidal carbon dioxide (EtCO_2_) for the duration of at least 30 s (8). “Sustained ROSC,” also referred to as a survived event, was defined as ROSC of at least 20 min duration (8). The supplemental variables “date and time of extubation,” “30 day survival,” “date of death after discharge,” and “cause of death after discharge” were not recorded on the CPR data form. If a patient suffered multiple arrests, only the first CPR event was described and analyzed for the present study. *Hospital* variables were not gathered on the individual patients' data forms since they describe the veterinary hospital at which cases are enrolled and CPR training, clinical practice, and equipment at our institution remained constant throughout the study period. Additional variables recorded that are not suggested by the veterinary Utstein-style CPR reporting guidelines included the qualification of the supervising clinician and number of people involved in CPR efforts.

Missing data from any category was retrospectively retrieved from the medical record. Definitions on the CPR data form generated in 2013 that were derived from 2004 human Utstein-style guidelines and that did not exactly match the categories published in the 2016 veterinary Utstein-style CPR reporting guidelines were retrospectively adjusted prior to data analysis. Severity scoring such as Acute Patient Physiologic and Laboratory Evaluation (APPLE) or pre-and post-arrest functional capacity by the Modified Glasgow Coma Scale (MGCS) was conducted retrospectively as previously described (18–20).

This manuscript is part of a two-manuscript series, factors associated with improved CPR outcomes are described in the companion paper.

### Statistical Analysis

All statistical analyses were performed using commercially available software (Prism 8.0, Graph Pad Software, La Jolla, CA, U.S.A.). Normality testing was performed on continuous variables using the Shapiro-Wilk test. None of the data sets were found to be normally distributed and non-parametric tests were used for further analyses. All continuous data is reported as median (range). Mann-Whitney U testing was used to compare variable distributions between patients achieving ROSC and those not achieving ROSC, between patients with a shockable and non-shockable first documented arrest rhythm, and to compare variables between the current study and a previous study conducted at the same facility prior to publication of RECOVER guidelines. Categorical data were compared between patients achieving ROSC and those not achieving ROSC and between patients with a shockable and non-shockable first documented arrest rhythm with the Fisher's exact test. Area under the receiver operating curve (AUC) analysis and 95% confidence interval (CI) for EtCO_2_ values measured in patients undergoing closed chest CPR only were calculated with the Wilson/Brown method. *P*-values of < 0.05 were considered statistically significant with *post-hoc* Bonferroni corrections applied to adjust for multiple comparisons where appropriate.

## Results

### Hospital Variables

During the study period, 177,125 dogs and cats presented to the hospital, and 46,874 were admitted to the hospital as inpatients. A total of 18,726 dogs and cats were presented to the small animal emergency service and the number of small animal cases anesthetized was 17,997. The total number of annual CPA events could not be determined since events without attempted CPR are not uniformly recorded in the electronic medical record. Time of day during which CPA occurred and training status of the CPR team leader were associated with patient outcome and are described in the accompanying manuscript.

### Outcome Variables

Out of 177,125 canine and feline visits to the hospital during the study period, a total of 219 CPR episodes were recorded (0.12%) in 172 dogs and 47 cats. Of the dogs and cats that underwent CPR, 44% of dogs (75/172) and 55% of cats (26/47) achieved ROSC. Seventeen percent of dogs (30/172) and 21% of cats (10/47) had ROSC ranging from 30 s to <20 min, 26% of dogs (45/172) and 34% of cats (16/47) had survived events (sustained ROSC that continued for ≥20 min) and 7% of dogs (12/172) and 19% of cats (9/47) survived to hospital discharge.

Of the animals that achieved ROSC for <20 min, 57% of dogs (17/30) and 80% of cats (8/10) were euthanatized. Of the 17 dogs euthanatized within 20 min of ROSC, 71% (12/17) were euthanatized due to poor prognosis, 12% (2/17) due to financial concerns, 12% (2/17) due to a combination thereof, and in one dog (6%) the reasons for euthanasia were not clear. Of the 8 cats euthanatized within 20 min of ROSC, 50% (4/8) were euthanatized due to poor prognosis and 50% (4/8) due to a combination of poor prognosis and owner financial constraints. The animals that did not achieve sustained ROSC and were not euthanatized (13 dogs and 2 cats) all experienced another CPA, of which none survived. The median duration of ROSC until euthanasia or rearrest was 4.75 (0.5–18) min.

Of the animals that had sustained ROSC (≥20 min), 27% of dogs (12/45) and 56% of cats (9/16) survived to hospital discharge. Of the animals that achieved sustained ROSC but that did not survive to hospital discharge, 58% of dogs (26/45) and 44% of cats (7/16) were euthanatized. The remaining 16% of dogs (7/45) suffered repeat CPA, none of which survived. Twenty-nine percent of dogs (13/45) that achieved sustained ROSC were alive at 24 h after CPA. Thereof, one dog (1/13, 8%) was euthanatized due to relapse of spontaneous tension pneumothorax that led to the first episode of CPA and the associated poor prognosis. The remaining 12 dogs (12/13, 92%) that were alive at 24 h post-CPA all survived to hospital discharge. Sixty-three percent of cats (10/16) that achieved sustained ROSC were alive at 24 h post-CPA. One cat (1/10, 10%) was euthanatized 4 days post-CPA due to poor prognosis associated with progressive multiorgan dysfunction. The remaining 9 cats (9/10, 90%) that were alive at 24 h post-CPA all survived to hospital discharge.

Compared to a study conducted at our institution prior to implementation of the RECOVER guidelines, overall (*p* = 0.12), canine (*p* = 0.07), and feline rates (*p* = 0.89) for achievement of any ROSC were not significantly different (3). Overall rates for sustained ROSC (*p* = 0.64), canine (*p* = 0.09), and feline (*p* = 0.12) sustained ROSC were also not significantly different. The overall rate of patients surviving to hospital discharge was not different between studies (*p* = 0.28) and neither was the canine survival to hospital discharge rate (*p* = 0.99). The feline survival to hospital discharge rate was statistically significantly higher in the present study compared to the study conducted prior to publication of the RECOVER guidelines (*p* = 0.0004).

### Animal Variables

Of the 172 dogs undergoing CPR, 83% (142/172) were purebred and 17% (30/172) were mixed breeds. Of the 47 cats, 51% (24/47) were domestic shorthair cats, 32% (15/47) were domestic medium or longhair cats, and 15% (7/47) were purebred cats. Breed could not be determined for one cat (1/47, 2%). Further information regarding gender distribution and weight of animals included in this study can be found in the accompanying manuscript investigating factors associated with improved CPR outcomes. Chest conformation of dogs and cats was not recorded in the present study.

Disease categories at the time of hospital admission are summarized in Supplemental Table 1. Both dogs and cats without ROSC most commonly presented to the hospital for medical, non-cardiac reasons, or DOA. Dogs and cats with ROSC commonly presented to the hospital for elective surgical procedures. Comorbidities at the time of CPA are summarized in Supplemental Table 2. The majority of patients had more than one comorbidity listed (median 1, range 0–7).

Forty-three percent of dogs that did not achieve ROSC (42/97) had no supportive, basic life support (BLS), or advanced life support (ALS) measures in place at the time of CPA. One or more CPR measures were already in place in 85% (64/75) of dogs that did achieve ROSC. Thirty three percent (7/21) of cats that did not achieve ROSC did not have any CPR measures in place at the time of CPA. One or more CPR measures were already in place in 77% of cats (20/26) that did achieve ROSC. An overview of all CPR measures in place at the time of CPA in all dogs and cats is provided in Supplemental Table 3 and associations between CPR measures already in place with patient outcome can be found in the accompanying manuscript. Medication constant rate infusions to support cardiovascular function were in place in 5% of dogs (5/97) not achieving ROSC and in 5% of dogs (4/75) achieving ROSC including norepinephrine in 3 dogs and dobutamine, vasopressin, and dopamine in one dog each not achieving ROSC, and norepinephrine (*n* = 1), and dopamine (*n* = 3) in dogs achieving ROSC. One dog not achieving ROSC received norepinephrine and vasopressin simultaneously. Medication constant rate infusions to support cardiovascular function were not in place in any cats that did not achieve ROSC and one cat that achieved ROSC had been receiving dopamine at the time of CPA.

Prearrest severity of illness as assessed using the canine and feline APPLE_fast_ scores could only retrospectively be calculated in 27 dogs (11 no ROSC, 16 ROSC) and 6 cats (2 no ROSC, 4 ROSC). Median APPLE_fast_ score in dogs not achieving ROSC was 29 (16–40) and in dogs achieving ROSC was 30 (17–33). In cats not achieving ROSC, median APPLE_fast_ score was 24.5 (23–26) and in cats achieving ROSC was 31.5 (14–37). Prearrest functional capacity as assessed by the modified Glasgow coma scale (MGCS) could be calculated for 11 dogs and 4 cats that achieved ROSC. The median score of dogs with ROSC was 16 (7–18). Median MGCS for cats achieving ROSC was 18 (17–18). No statistical associations with patient outcome were performed due to the low numbers of patients in which MGCS and Apple_fast_ scores were available.

### Arrest Variables

CPA was witnessed in 66% of dogs (64/97) not achieving ROSC and in 89% (67/75) of dogs achieving ROSC. In cats, CPA was a witnessed event in 52% (11/21) of patients not achieving ROSC and in 85% (22/26) of cats that achieved ROSC.

Suspected causes of CPA are summarized in Table 1. A total of 43 animals (20%) had more than one suspected cause of arrest listed. The suspected cause of CPA remained unknown in the majority (*n* = 68, 31%) of animals, and suffering CPA due to an unknown cause was significantly associated with not achieving ROSC (*p* = 0.0012). Suffering CPA due to a drug overdose or toxicity was significantly associated with ROSC (*p* < 0.0001).

**Table 1:** Suspected cause of CPA in 172 dogs and 47 cats undergoing CPR.

	**Dogs** ***n*** **(%)**		**Cats** ***n*** **(%)**		**Dogs and Cats**
	**No ROSC (*n* = 97)**	**ROSC (*n* = 75)**	**No ROSC (*n* = 21)**	**ROSC (*n* = 26)**	***P*-value**
Arrhythmia	3 (3)	5 (7)	1 (5)	0 (0)	0.74
Respiratory failure	14 (14)	19 (25)	4 (19)	6 (23)	0.09
Heart failure	10 (10)	10 (13)	2 (10)	2 (8)	0.83
Trauma	13 (13)	3 (4)	0 (0)	0 (0)	0.04
Hemorrhage	7 (7)	8 (11)	2 (10)	3 (12)	0.48
Hypovolemia	12 (12)	2 (3)	0 (0)	1 (4)	0.06
Brain disease	5 (5)	8 (11)	3 (14)	3 (12)	0.34
Severe sepsis/septic shock	3 (3)	7 (9)	0 (0)	1 (4)	0.12
MODS	1 (1)	0 (0)	0 (0)	1 (4)	1.0
Metabolic/electrolyte	8 (8)	8 (11)	1 (5)	6 (23)	0.19
Toxicity/overdose^*^	1 (1)	8 (11)	0 (0)	8 (31)	<0.0001
Unknown^*^	37 (38)	16 (21)	11 (52)	4 (15)	0.0012

*CPA, cardiopulmonary arrest; CPR, cardiopulmonary resuscitation; MODS, multiorgan dysfunction syndrome; ROSC, return of spontaneous circulation*.

* *Denotes CPA causes significantly different between patients with and without ROSC after Bonferroni correction*.

In-hospital CPA was more common in both dogs and cats in this study compared to OHCA. The majority of dogs and cats receiving CPR in this study were categorized as emergency room outpatients at the time of CPA (99/219, 45%). ICU inpatients were the second most common group of animals receiving CPR (57/219, 26%) The specific location of IHCA is summarized in Supplemental Table 4. Location of IHCA could not be determined in 2 dogs achieving ROSC.

Median time to CPR initiation after CPA recognition in the present study was 12 s (immediate−21 min) in patients with ROSC and 12 s (0.2–13 min) in patients without ROSC, which was not significantly different (*p* = 0.5). The time to CPR in patients with and without ROSC was not significantly different in this study compared to a study at the same facility prior to publication of the RECOVER guidelines (*p* = 0.33).

**Figure 1:**
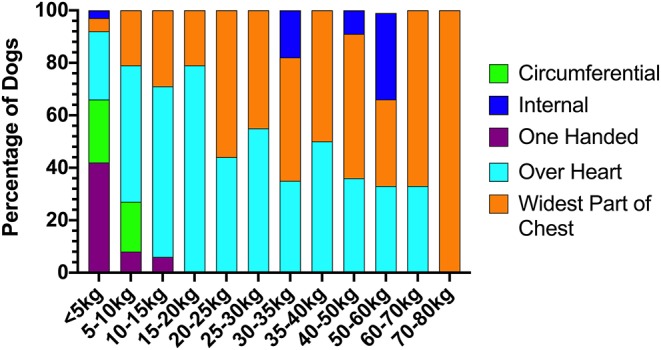
Chest compression techniques applied to dogs during CPR according to body weight. CPR, cardiopulmonary resuscitation.

**Figure 2:**
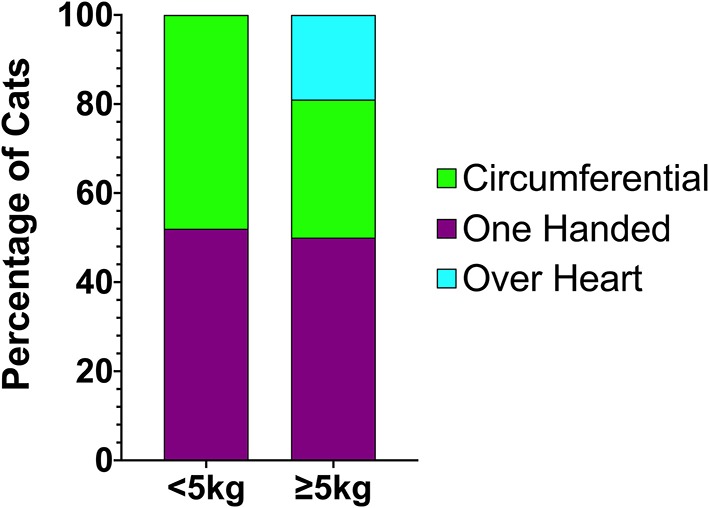
Chest compression techniques applied to cats during CPR according to body weight. CPR, cardiopulmonary resuscitation.

RECOVER guideline compliance for BLS and ALS execution in this study was variable. Table 2 summarizes the percentage of compliance with key aspects of BLS and ALS performance. All closed chest compressions were performed with animals in lateral recumbency. Hand positioning during chest compressions was reported in all animals except for two dogs. In 9% (15/172) of dogs and 15% (7/47) of cats, multiple chest compression techniques were reported. In both dogs with and without ROSC, chest compressions were most commonly performed with both hands positioned directly over the heart (55% 53/96, and 45% 33/74 respectively). The second most commonly employed technique was compressions over the widest part of the thorax (32/96, 33%; 23/74, 31%). In cats, a one-handed compression technique was most commonly employed (28/47, 60%). The second most common technique in cats was using a two hands circumferential technique (22/47, 47%). Hand positions during CPR according to patient body weight are summarized in Figures 1, 2.

**Table 2:** Compliance with RECOVER clinical guideline recommendations for key aspects of BLS and ALS in 172 dogs and 47 cats undergoing CPR.

**Variable**	**Guideline recommendation**	**Compliance *n* (%)**	**Clinically acceptable alternative**	**Compliance** ***n* (%)**
**BLS**				
Chest compression rate	100–120 per minute	158/209 (76)	100–150 per minute	182/209 (87)
Intubation	All patients	211/219 (96)	Mouth to snout ventilation only	0/219 (0)
Ventilation rate	10 per minute	92/184 (50)	8–12 per minute	137/184 (74)
**ALS**				
EtCO_2_ monitoring	All patients	129/219 (59)	–	–
ECG monitoring	All patients	207/219 (95)	–	–
IVC placement	All patients	190/193 (98)^*^	IO catheter	1/193 (<1)
Epinephrine administration	All patients with diagnosed non-shockable arrest rhythm	66/135 (49)	Patients with non-shockable or unknown arrest rhythm	197/219 (90)
Epinephrine dose	0.01 mg/kg	124/197 (63)	0.01–0.1 mg/kg	154/197 (78)
Defibrillation administration	All patients with shockable arrest rhythm	42/55 (76)	Precordial thump	0/55 (0)

*ALS, advanced life support; BLS, basic life support; CPR, cardiopulmonary resuscitation, ECG, electrocardiogram; EtCO_2_, end tidal carbon dioxide; IVC, intravenous catheter; IO, intraosseous; mg/kg, milligram per kilogram; RECOVER, Reassessment Campaign on Veterinary Resuscitation*.

* *Extrapolated from route of epinephrine administration*.

Median chest compression rates for dogs not achieving ROSC were 120 (70–160) per minute and 120 (60–225) per minute for dogs achieving ROSC. Median chest compression rate in cats that did not achieve ROSC was 150 (120–200) per minute and in cats that did achieve ROSC was 120 (80–190) per minute. Median chest compression rates between animals with and without ROSC were not significantly different (*p* = 0.66). At a median of 120 (60–225) compressions per minute, chest compression rates in this study were significantly higher than chest compression rates employed at our facility prior to introduction of a RECOVER based CPR approach [median of 105 (65–230) compressions per minute (*p* < 0.0001)].

EtCO_2_ was monitored in 59% (129/219) of patients and a reading could be obtained in 84% (109/129) thereof. Excessive airway fluid was noted in 45% (9/20) of patients in which an EtCO_2_ reading could not be obtained. In dogs and cats that did not achieve ROSC, the median EtCO_2_ was 15 (0–54) mmHg and in those that achieved ROSC was 23 (9–61) mmHg, which was significantly different (*p* = 0.0004). EtCO_2_ demonstrated a fair accuracy at discriminating between patients undergoing closed chest CPR that did and did not achieve ROSC with AUC (95% CI) of 0.715 (0.618–0.811), *p* = 0.0002. The optimal cutoff for the performance of EtCO_2_ as an outcome discriminator was 16.5 mmHg with a sensitivity (95% CI) of 75% (60–86%) and specificity (95% CI) of 64% (52–75%).

Endotracheal intubation was performed in 99% (96/97) of dogs not achieving ROSC, and 93% (70/75) of dogs achieving ROSC. Of cats not achieving ROSC, 100% (21/21) were intubated, as were 92% (24/26) of cats that achieved ROSC. A minority of dogs not achieving ROSC (7%, 7/97) and dogs achieving ROSC (12%, 9/75) were already intubated at the time of CPA. One cat that did not achieve ROSC (5%, 1/21) and 11% (3/26) of cats that achieved ROSC were already intubated at the time of CPA. Mouth to snout ventilation was performed until endotracheal intubation could be achieved in 1 dog achieving ROSC (1%, 1/75) and 1 dog not achieving ROSC (1%, 1/97). Mouth to snout ventilation was never employed in cats. Ventilation was never provided using a tight-fitting face mask in either species. Median time to intubation was not different (*p* = 0.064) between patients not achieving ROSC [1 (<0.5–14) min] and in patients achieving ROSC [1 (<0.5–15) min]. The median ventilatory rate in patients not achieving ROSC was 10 (5–40) per minute and in patients achieving ROSC was 10 (5–60), *p* = 0.07) per minute. The distribution of ventilatory rates recorded in this study [10 (5–60) per minute] was significantly different compared to that recorded in our previous study [11 (5–60) per minute, *p* < 0.0001].

Cardiac rhythms were monitored during CPR efforts in 95% of dogs (161/172) and 98% of cats (46/47); and in 32% of dogs (55/172) and 13% of cats (6/47), an ECG was already in place at the time of CPA. Median time to first rhythm diagnosis was 3 (0.5–13) min in dogs and 3.5 (1–10) min in cats. A rhythm diagnosis at the time of CPA or during CPR efforts was available for 77% of dogs (124/161) and 70% of cats (32/46). In the remainder of the animals, the first obtained rhythm diagnosis occurred only at the time of ROSC. Individual rhythm diagnoses obtained at the time of CPA or during CPR are summarized in Table 3.

**Table 3:** First diagnosed cardiac rhythm at the time of CPA or during CPR in 124 dogs and 32 cats with ECG diagnoses recorded.

	**Dogs *n* (%)**	**Cats *n* (%)**
**NON-SHOCKABLE RHYTHM**	**98 (79)**	**27 (84)**
Asystole	65 (66)	19 (70)
Pulseless electrical activity	33 (34)	8 (30
**SHOCKABLE RHYTHM**	**26 (21)**	**5 (16)**
Pulseless ventricular tachycardia	4 (15)	0 (0)
Ventricular fibrillation	22 (85)	5 (100)

*CPA, cardiopulmonary arrest; CPR, cardiopulmonary resuscitation; ECG, electrocardiogram*.

*The bold values represent the sum for all shockable rhythms = sum of Pulseless ventricular tachycardia plus ventricular fibrillation and the sum of all non-shockable rhythms = sum of asystole plus PEA*.

The presence of a shockable first documented arrest rhythm vs. a non-shockable one was not statistically significantly associated with achieving ROSC (*p* = 0.12) when analyzing canine and feline rhythms collectively. When comparing rhythms of dogs and cats achieving ROSC and not achieving ROSC separately, the presence of a shockable first documented rhythm in dogs was significantly associated with ROSC (*p* = 0.02), whereas in cats, there was no association of first documented rhythm and ROSC (*p* = 0.63). Median number of comorbidities present at the time of CPA (categories of comorbidities listed in Supplemental Table 2) in patients with a shockable first documented rhythm were 1 (0–7), and median number of comorbidities present at the time of CPA in patients with a non-shockable first documented arrest rhythm were 2 (0–8), which was not significantly different (*p* = 0.17).

Throughout the course of CPR, 22% (22/98) of all non-shockable rhythms in dogs became shockable, as did 7% (2/27) of initially non-shockable rhythms in cats. The development of a subsequent shockable rhythm compared to never having a shockable rhythm was not associated with ROSC (*p* = 0.3).

Thirty-seven dogs (21/26 with initial shockable rhythms and 16/22 with subsequent shockable rhythms) and 5 cats were defibrillated at least once. Median time from start of CPR to defibrillation was 8 (1–23) min and initial defibrillation was successful in 52% of cases (22/42), to a perfusing rhythm in 41% (9/22) and to a non-perfusing rhythm in 59% (13/22).

Defibrillation in patients with an initially shockable rhythm was successful in 71% (17/24) of cases, whereas defibrillation in patients developing a shockable rhythm during CPR was only successful in 28% (5/18) of cases. This was statistically significantly different (*p* = 0.0116).

Information on epinephrine administration timing, time to first arrest rhythm, and ECG diagnosis was available in 135 animals. Administration of epinephrine occurred prior to obtaining a rhythm diagnosis in 51% of patients (69/135). In 19% of these animals (13/69) the first documented arrest rhythm subsequently diagnosed was shockable and in 16% of animals (11/69), the first documented arrest rhythm was non-shockable but converted to a shockable rhythm throughout the course of CPR. This was not different to the numbers of initial non-shockable rhythms that converted to shockable rhythms in 15% (10/66) of animals in which epinephrine was administered only after confirming a non-shockable rhythm (*p* > 0.99). The proportion of unsuccessfully defibrillated to successfully defibrillated patients in those that received epinephrine prior to rhythm diagnosis (50%, 9/18) and those that received epinephrine after rhythm diagnosis was not significantly different (53%, 9/17; *p* > 0.99). Patients with initial or subsequent shockable arrest rhythms were administered lidocaine in 5 cases, amiodarone in 2 cases and magnesium sulfate in 3 cases.

Ninety percent of patients in this study received epinephrine as part of ALS efforts (197/219), whereas vasopressin was only administered to 5% of patients (11/219). Atropine was given in addition to epinephrine in 81% of patients (177/219), atropine alone was given in 4% (9/219) of patients. Reversal agents were used in 5% of cases (11/219), two patients received two reversal agents. A total of 7 patients received naloxone, 4 patients received flumazenil, and two patients received atipamezole. Of the 11 patients, 4 received reversal agents due to arrest under general anesthesia, constituting 25% (4/16) of the general anesthesia patient population. Of those 4 patients, 2 received naloxone only and 2 received both naloxone and flumazenil. Sodium bicarbonate therapy was instituted in 4% (9/219) of cases. Forty-five percent of patients (98/219) received isotonic crystalloids, 2% (4/219) synthetic colloids, and 10% (21/219) 7.2% sodium chloride during CPR efforts. Blood products were administered to 5% of patients during CPR, packed red blood cells to 4% (8/219) and plasma products to 1% (3/219).

A total of 222 CPR complications were reported in 143 patients (65%) and multiple complications occurred in 55 patients (32%). Complications included difficulties with intubation (62), ETT dislodgement (12), excessive airway fluid hampering ventilation (40), loss of IVC (9), loss of ECG leads (22), defibrillator malfunction (4), inability to deliver shock (8), safety concerns precluding shock delivery (1), and incorrect drug dosages (64). Out of the incorrect drug dosages, atropine was administered at too high a dose (>0.1 mg/kg) in 3 cases, at too low a dose (<0.02 mg/kg) in 18 cases, and epinephrine was administered at too high a dose (>0.1 mg/kg) in 10 and at too low a dose (<0.01 mg/kg) in 33 cases.

## Discussion

In this study, 44% of dogs and 55% of cats achieved initial ROSC, which is comparable to the outcomes reported by the previous study at our institution and a further improvement in patient outcomes could not be confirmed since publication of the RECOVER guidelines (3). Overall, BLS was conducted very similarly before and after publication of the RECOVER guidelines. Even though chest compression rates were significantly higher and ventilatory rates were significantly lower in this study, the rates applied in both studies were within the range recommended by the RECOVER guidelines. In accordance with previous veterinary studies, the majority of patients had a first documented CPR rhythm that was non-shockable and only a minority of patients required electrical defibrillation (2, 3, 21). The majority of patients had epinephrine and atropine administered during CPR efforts but in approximately half of the cases, this did not occur in accordance with the RECOVER guidelines after ECG diagnosis of a non-shockable rhythm, but as soon as IV access had been established.

### Hospital Variables

With approximately 39,000 cases admitted to the hospital per year, the annual small animal case load increased approximately 30% in this study period compared to a previous study conducted at the same institution (3). In our previous study, 0.2% of patients visiting the hospital underwent CPR, which has remained comparable in the present study with 0.12% of patients suffering CPA and undergoing CPR efforts (3). It is unknown how many patients during this study's timeframe suffered CPA and did not undergo resuscitative efforts and whether this may have been due to owners' wishes or due to clinician perceived futility. As not every owner wishes for their pet to undergo CPR, this represents a selection bias and it remains unknown how successful CPR could be if it was offered to every patient suffering CPA.

### Outcome Variables

The rates for any ROSC and sustained ROSC in the current study are not significantly different from those of a previous study performed at the same institution prior to publication of the RECOVER guidelines (1, 3). Furthermore, they remain comparable to previous veterinary studies reporting ROSC in 28–60% of dogs and 42–57% of cats, and to reports in people of ROSC rates of up to 53% for IHCA cardiac arrest (2, 3, 5–7, 22). In this study, ROSC was defined as return of effective circulation for at least 30 s, as defined by the Utstein criteria (8). Previous veterinary studies have defined ROSC as return of spontaneous circulation for variable periods of time and this limits the relevance of direct comparison of results. ROSC rates in excess of 40% in veterinary medicine have only been reported in recent years, likely representing an improved understanding of CPR techniques and the adoption of standardized approaches to CPR (3, 4, 7, 16).

Recently, a study conducted at a Japanese small animal nighttime hospital was able to demonstrate significantly improved ROSC rates after employing a RECOVER based approach to CPR (4). In contrast to the Japanese study, ROSC rates at our facility prior to 2012 were already high, which is likely why a further increase could not be demonstrated. This difference in pre-RECOVER ROSC rates could be a result of our institution's earlier awareness of the importance of a standardized approach to CPR and adoption of many of the American Heart Association 2010 guidelines, prior to the development of the RECOVER initiative (23).

The euthanasia and rearrest rates of dogs and cats following ROSC in this study were similar to our previous study and likely reflect the severity of the primary disease processes present in addition to medical concerns related to post-CPR complications (3). Overall, until 24 h post-arrest, animals are more likely to get euthanatized than to suffer rearrest. This indicates that euthanasia remains a major confounding factor in assessing our post-arrest care capabilities and factors associated with intermediate- and long-term CPR outcomes. Further studies of the post-CPA period are needed to gather more information on factors associated with long-term survival after suffering CPA.

Even though veterinary ROSC rates in North America have become comparable to ROSC rates in people, the rate of survival to hospital discharge in veterinary medicine overall remains significantly lower (3, 7, 24, 25). Compared to the study previously performed at our institution, feline survival to hospital discharge rates are significantly higher in the current study, reaching 19% compared to the previously reported 3% (3). Even though not statistically significant, cats in the present study tended to have slightly higher ROSC rates than dogs (55% vs. 44%, *p* = 0.15), whilst a similar percentage of patients were euthanatized per species, leaving more cats to survive to hospital discharge. The body conformation of cats likely makes chest compressions more effective than in most dogs and may account for the higher survival rate of cats in this study. Survival to hospital discharge is also dependent on the severity of the underlying illness and it remains possible that the cats included in this study were less severely ill than dogs. In our accompanying manuscript, being a cat was associated with increased odds for survival to hospital discharge even after controlling for being under general anesthesia at the time of arrest, making a general anesthesia related CPA as an explanation for higher survival unlikely. Illness severity scoring and prearrest functional capacity assessment could only be performed in a minority of patients and therefore differences in prearrest illness severity and patient neurologic function remain factors that could explain the higher feline survival to hospital discharge rates.

### Arrest Variables

Cardiopulmonary arrest in patients with an unknown underlying etiology was associated with lower likelihood of ROSC, whereas CPA due to drug overdose or toxicity was associated with achieving ROSC. We were not able to confirm the previously reported lower likelihood of ROSC in patients suffering CPA due to hemorrhage, shock, multiorgan dysfunction syndrome, or neurologic disease (2, 3). Patients experiencing CPA due to drug overdoses or toxicities in this study included the patients arresting under general anesthesia, a patient population that has previously been shown to have improved odds of achieving ROSC most likely due to a better overall health status compared to patients with other precipitating causes of arrest (2, 3, 5).

The median chest compression rates in our study were not different between patients with and without ROSC and fall into the rates recommended by the RECOVER guidelines (1). Even though chest compression rates employed at our institution are significantly higher since publication of the RECOVER guidelines, both the rates prior to and after their implementation fall within the recommended range and the difference is unlikely to be clinically significant (1).

EtCO_2_ monitoring has been recommended to assess the efficacy of chest compressions and predict CPR outcomes in people and the RECOVER initiative has adopted the recommendation of its routine monitoring during CPR (1, 26, 27). Compared to a previous study conducted at our institution, the use of EtCO_2_ has increased from being uncommon to being monitored in >50% of patients since publication of the RECOVER guidelines (3). This rate of use falls within the previously reported rates of 30–40% in general practice and >70% by specialists (28).

Patients achieving ROSC in our study had significantly higher median EtCO_2_ than patients that did not achieve ROSC and an EtCO_2_ cutoff of 16.5 mmHg was a fair discriminator of achieving ROSC. This value is similar to values reported in a previously conducted veterinary study that was able to demonstrate higher ROSC rates for dogs and cats if an EtCO_2_ of > 15 mmHg and > 20 mmHg can be achieved during CPR respectively and a more recent study that showed that EtCO_2_ of ≥18 mmHg after 2 min of CPR is a strong predictor of ROSC (2, 16). Furthermore, it is similar to a study in people in which no patients with a maximum EtCO_2_ < 10 mmHg could be successfully resuscitated and that showed that an EtCO_2_ of ≥ 14.3 mmHg after 20 min of CPR predicted ROSC with 100% sensitivity and specificity (29). In up to 16% of cases in which a capnometer was used, no reading could be obtained either due to equipment malfunction or due to clogging of the mainstream capnometer with airway secretions. This is a problem that has previously been reported and it is possible that side stream capnometers would function more reliably in patients expected to have excessive airway secretions during CPR (16).

The majority of patients in the present study were intubated as recommended by the RECOVER guidelines and the mean time to intubation was short at 1 min in both animals with and without ROSC. Difficulty intubating patients was reported in 28% of patients in the present study. Most commonly, intubation was perceived to be difficult if it took up to 1 min or longer to achieve or if it had to be attempted more than once due to initial esophageal intubation. Despite the reported difficulties with intubation, successful endotracheal intubation was achieved in the majority of patients. The range of time to intubation was wide in this study and mouth to snout ventilation prior to endotracheal intubation was only performed in two dogs and no cats. It is possible that consideration of alternative ventilation techniques when endotracheal intubation was difficult or delayed could have been of benefit.

The frequency and nature of the first documented ECG diagnosis in this study was similar to previous studies although the rate of pulseless electrical activity in this study was higher than in earlier reports (2, 3, 21). In people, the presence of an initial shockable arrest rhythm is associated with a better patient outcome compared to initial arrest rhythms that are non-shockable and it has been shown that patients with shockable arrest rhythms are less likely to suffer comorbidities such as pulmonary or renal dysfunction that would further negatively impact their outcome (30). This is the first veterinary study that was also able to demonstrate a better outcome of canine patients with a first documented rhythm that is shockable compared to a first documented non-shockable rhythm. In cats, we were not able to demonstrate an improved outcome in patients with a shockable first documented arrest rhythm but given the small number of patients that were defibrillated this may represent a type II error.

In the current study, defibrillation attempts were only successful in 52% of animals, which is not dissimilar to a previous veterinary study reporting a 63% success rate, but lower than the success rates of single shock defibrillation reported in human adult cardiac arrests (3, 31). In almost 30% of defibrillation events in this study, defibrillator malfunction or user inability to deliver the shock was reported and these delays in defibrillation could have reduced the success rate achieved. The human literature suggests that the outcome associated with a shockable rhythm may be different if it is the initial arrest rhythm vs. a rhythm that develops during CPR (30, 32, 33). Approximately half of the animals defibrillated in this study were treated for a shockable rhythm that developed during CPR making successful defibrillation less likely (30).

Human CPR guidelines recommend the use of epinephrine every 3–5 min during CPR for both shockable and non-shockable rhythms, the RECOVER guidelines currently only recommend its use after the diagnosis of a non-shockable rhythm (1, 34–36). In the present study, the adherence to this recommendation was poor and epinephrine was administered to 51% of patients as soon as IV access was achieved and prior to obtaining an ECG rhythm diagnosis. No negative consequences such as a pro-arrhythmogenic effects were evident in our study and no difference in outcome was found when compared to animals in which epinephrine was only given after an ECG diagnosis was made.

The reported CPR complication rate in this study is higher than the complication rate reported in a previous study at our institution (3). This may be a product of the new data form used for the current study which included a list of complications to check and complications did not have to be listed as a free text. It is possible that prompting clinicians to identify complications on the data form may have increased their recognition. Endotracheal tube dislodgement and incorrect drug dosages remained frequent complications, however due to the different methods of complication recording, it remains unclear if the incidence has changed.

## Limitations and Conclusions

This study has several limitations despite its prospective nature. While clinicians leading CPR efforts in this study were all familiar with the RECOVER guidelines, BLS and ALS execution were not standardized by protocol. Since clinicians filled out CPR data forms after conclusion of CPR efforts, it remains possible that the not all data was captured accurately and that reported results were influenced by theoretical knowledge of the RECOVER guidelines.

Ideal compliance with RECOVER guideline recommendations was variable for different BLS and ALS tasks in the current study. In human medicine, it has been well established, that the true benefit of practice guidelines can only be assessed against the background of guideline adherence and it has been shown that higher compliance with CPR guidelines leads to significantly higher quality of patient care, ROSC, and survival to hospital discharge rates (37–39). It therefore remains unclear what the benefit of the RECOVER guidelines for patient outcome would be if they were adhered to perfectly. Furthermore, assessment of the appropriateness of BLS efforts was limited by the fact that chest conformation was not recorded in this study. As a significant proportion of patients in this study arrived at the hospital DOA, physical examination and laboratory parameters prior to CPA were unavailable. Unfortunately, this precluded calculation of illness severity scores such as the APPLE_fast_ score in many cases and it remains possible that the pre-CPA illness severity of this study population does not compare to the study population of the previous study conducted at our institution.

While most aspects of CPR practice, overall ROSC rates, and survival to hospital discharge rates in this study performed post-publication of the RECOVER guidelines were not significantly different compared to outcomes at the same institution prior to their publication, a significantly higher proportion of cats survived to hospital discharge. We cannot exclude that the feline patient population in this study had a lower illness severity than cats in previous studies resulting in the better apparent outcomes. Future prospective studies with more rigorous recording methods of CPR events would be of benefit to better determine factors that may influence patient outcome.

## Data Availability Statement

The raw data generated for this study can be made available to any qualified researcher upon request to the corresponding author.

## Ethics Statement

Ethical review and approval was not required for the animal study because the study was observational in nature and no interventions took place that would have altered the treatment of animals included in this study. Written informed consent for participation was not obtained from the owners because the study was purely observational and did not alter the standard of care in patients undergoing CPR. Owner consent to perform CPR was obtained either verbally (in an emergency situation) or written at patient admission to the hospital.

## Author Contributions

KH and SE designed the study and generated the CPR questionnaire and critically revised the manuscript. SH compiled and analyzed the data and wrote the manuscript. Results were interpreted by SH, KH, and SE. All authors read and approved the final manuscript.

### Conflict of Interest

The authors declare that the research was conducted in the absence of any commercial or financial relationships that could be construed as a potential conflict of interest.

## Abbreviations

ALS: Advanced life support
APPLE: Acute Patient Physiologic and Laboratory Evaluation
AUC: Area under the receiver operating curve
BLS: Basic life support
CI: Confidence interval
CPA: Cardiopulmonary arrest
CPR: Cardiopulmonary resuscitation
DOA: Dead on arrival
ECG: Electrocardiogram
EtCO_2_: End Tidal Carbon Dioxide
ICU: Intensive Care Unit
IHCA: In-hospital cardiopulmonary arrest
MGCS: Modified Glasgow Coma Scale
OHCA: Out-of-hospital cardiopulmonary arrest
RECOVER: Reassessment Campaign on Veterinary Resuscitation
ROSC: Return of spontaneous circulation.
