# Supplementary material for: Prospective Evaluation of Cardiopulmonary Resuscitation Performed in Dogs and Cats According to the RECOVER Guidelines. Part 2: Patient Outcomes and CPR Practice Since Guideline Implementation
Source: Front Vet Sci. 2019 Dec 10;6:439. doi: 10.3389/fvets.2019.00439 (PMC6914737; doi:10.3389/fvets.2019.00439)
Supplement: Supplementary file 1 [file Table_1.docx]

**Supplemental Table 1:** Disease categories at admission to the hospital and rates of ROSC in 172 dogs and 47 cats undergoing CPR

|  | **Dogs n (%)** | | **Cats n (%)** | |
| --- | --- | --- | --- | --- |
|  | **No ROSC (n=97)** | **ROSC (n=75)** | **No ROSC (n=21)** | **ROSC (n=26)** |
| Medical cardiac | 8 (8) | 8 (11) | 2 (10) | 1 (4) |
| Medical | 37 (38) | 35 (47) | 5 (24) | 13 (50) |
| Surgery elective | 7 (7) | 14 (19) | 3 (14) | 5 (19) |
| Surgery emergent | 3 (3) | 2 (3) | 0 (0) | 0 (0) |
| Trauma | 3 (3) | 3 (4) | 2 (10) | 2 (8) |
| DOA | 39 (40) | 13 (17) | 9 (43) | 5 (19) |
| Unknown | 0 (0) | 0 (0) | 0 (0) | 0 (0) |

CPR, Cardiopulmonary resuscitation; DOA, Dead on arrival; ROSC, Return of spontaneous circulation.
