# Supplementary material for: Prospective Evaluation of Cardiopulmonary Resuscitation Performed in Dogs and Cats According to the RECOVER Guidelines. Part 2: Patient Outcomes and CPR Practice Since Guideline Implementation
Source: Front Vet Sci. 2019 Dec 10;6:439. doi: 10.3389/fvets.2019.00439 (PMC6914737; doi:10.3389/fvets.2019.00439)
Supplement: Supplementary file 2 [file Table_2.docx]

**Supplemental Table 2:** Comorbidities present at the time of CPA in 172 dogs and 47 cats undergoing CPR

|  | **Dogs n (%)** | | **Cats n (%)** | |
| --- | --- | --- | --- | --- |
|  | **No ROSC (n=97)** | **ROSC (n=75)** | **No ROSC (n=21)** | **ROSC (n=26)** |
| Arrhythmias | 8 (8) | 14 (19) | 0 (0) | 0 (0) |
| Previous CHF | 3 (3) | 2 (3) | 0 (0) | 1 (4) |
| Current CHF | 6 (6) | 7 (9) | 2 (10) | 1 (4) |
| Pericardial disease | 3 (3) | 2 (3) | 0 (0) | 0 (0) |
| Hypotension | 18 (18) | 25 (33) | 5 (24) | 6 (23) |
| Respiratory | 20 (20) | 27 (36) | 4 (19) | 4 (15) |
| Pneumonia | 7 (7) | 9 (12) | 0 (0) | 1 (4) |
| Renal insufficiency | 10 (10) | 12 (16) | 1 (5) | 4 (15) |
| Hepatic insufficiency | 8 (8) | 10 (13) | 0 (0) | 1 (4) |
| CNS disease | 18 (18) | 20 (27) | 4 (19) | 2 (8) |
| SIRS | 9 (9) | 8 (11) | 0 (0) | 1 (4) |
| Sepsis | 8 (8) | 11 (15) | 0 (0) | 0 (0) |
| Infectious | 11 (11) | 7 (9) | 2 (10) | 0 (0) |
| Diabetes mellitus | 1 (1) | 2 (3) | 0 (0) | 0 (0) |
| Metabolic | 23 (23) | 24 (32) | 5 (24) | 12 (46) |
| Malignancy | 14 (14) | 5 (7) | 3 (14) | 5 (19) |
| Trauma | 13 (13) | 4 (5) | 0 (0) | 0 (0) |
| Envenomation | 0 (0) | 0 (0) | 0 (0) | 0 (0) |
| Postoperative | 0 (0) | 0 (0) | 1 (5) | 1 (4) |
| None | 7 (7) | 5 (7) | 0 (0) | 2 (8) |
| Unknown | 10 (10) | 3 (4) | 5 (24) | 5 (19) |

CHF, Congestive heart failure; CNS, Central nervous system; CPA, Cardiopulmonary arrest; CPR, Cardiopulmonary resuscitation; ROSC, Return of spontaneous circulation; SIRS, Systemic inflammatory response syndrome.
