# Supplementary material for: Prospective Evaluation of Cardiopulmonary Resuscitation Performed in Dogs and Cats According to the RECOVER Guidelines. Part 2: Patient Outcomes and CPR Practice Since Guideline Implementation
Source: Front Vet Sci. 2019 Dec 10;6:439. doi: 10.3389/fvets.2019.00439 (PMC6914737; doi:10.3389/fvets.2019.00439)
Supplement: Supplementary file 3 [file Table_3.docx]

**Supplemental Table 3:** Monitoring equipment and supportive measures already in place at the time of CPA in 172 dogs and 47 cats undergoing CPR

|  | **Dogs n (%)** | | **Cats n (%)** | |
| --- | --- | --- | --- | --- |
|  | **No ROSC (n=97)** | **ROSC (n=75)** | **No ROSC (n=21)** | **ROSC (n=26)** |
| Intubated | 9 (9) | 8 (11) | 1 (5) | 2 (8) |
| Supplemental O_2_ | 26 (27) | 30 (40) | 4 (19) | 9 (35) |
| Mechanical ventilation | 5 (5) | 5 (7) | 0 (0) | 2 (8) |
| ECG | 28 (29) | 27 (36) | 3 (14) | 3 (12) |
| EtCO_2_ | 6 (6) | 6 (8) | 0 (0) | 1 (4) |
| NIBP | 8 (8) | 14 (19) | 1 (5) | 3 (12) |
| IBP | 6 (6) | 5 (7) | 0 (0) | 0 (0) |
| Peripheral IVC | 49 (51) | 56 (75) | 13 (62) | 16 (62) |
| Central IVC | 16 (16) | 17 (23) | 0 (0) | 6 (23) |
| Arterial catheter | 7 (7) | 6 (8) | 0 (0) | 1 (4) |
| IO catheter | 0 (0) | 0 (0) | 0 (0) | 0 (0) |

CPA, Cardiopulmonary arrest; CPR, Cardiopulmonary resuscitation, ECG, Electrocardiogram; EtCO2, End tidal carbon dioxide monitoring; IBP, Invasive blood pressure measurements; IO, Intraosseous; IVC, Intravenous catheter; NIBP, Non-invasive blood pressure measurements; O_2,_ Oxygen; ROSC, Return of spontaneous circulation.
