# Supplementary material for: Prospective Evaluation of Cardiopulmonary Resuscitation Performed in Dogs and Cats According to the RECOVER Guidelines. Part 2: Patient Outcomes and CPR Practice Since Guideline Implementation
Source: Front Vet Sci. 2019 Dec 10;6:439. doi: 10.3389/fvets.2019.00439 (PMC6914737; doi:10.3389/fvets.2019.00439)
Supplement: Supplementary file 4 [file Table_4.docx]

**Supplemental Table 4:** Location of CPA in 172 dogs and 47 cats undergoing CPR

|  | **Dogs n (%)** | | **Cats n (%)** | |
| --- | --- | --- | --- | --- |
|  | **No ROSC (n=97)** | **ROSC (n=75)** | **No ROSC (n=21)** | **ROSC (n=26)** |
| Out of Hospital | 35 (36) | 11 (15) | 8 (38) | 5 (19) |
| In Hospital: | 62 (64) | 64 (85) | 13 (62) | 21 (81) |
| ER | 18 (29) | 17 (27) | 4 (31) | 5 (24) |
| Consult room | 0 (0) | 0 (0) | 0 (0) | 0 (0) |
| ICU | 19 (31) | 26 (41) | 2 (15) | 9 (43) |
| Patient care wards | 15 (24) | 5 (8) | 4 (31) | 0 (0) |
| Anesthesia induction | 0 (0) | 0 (0) | 0 (0) | 1 (5) |
| Operating room | 2 (3) | 2 (3) | 0 (0) | 0 (0) |
| Procedure room | 6 (10) | 5 (8) | 1 (8) | 5 (24) |
| Waiting room | 1 (1) | 1 (1) | 1 (5) | 1 (4) |
| Other | 1 (1) | 6 (8) | 1 (5) | 0 (0) |

CPA, Cardiopulmonary arrest; CPR, Cardiopulmonary resuscitation; ER, Emergency room; ICU, Intensive care unit; ROSC, Return of spontaneous circulation. Other include CPA while walking inpatients outside (n=3), post-anesthesia recovery room (n=4), and in the student surgery laboratory facility (n=1). The location of in-hospital CPA could not be determined for 2 dogs achieving ROSC.
